# Effect of electrolyte flow on a gas evolution electrode

**DOI:** 10.1038/s41598-021-84084-1

**Published:** 2021-02-25

**Authors:** Soufiane Abdelghani-Idrissi, Nicolas Dubouis, Alexis Grimaud, Philippe Stevens, Gwenaëlle Toussaint, Annie Colin

**Affiliations:** 1 ESPCI Paris, PSL Research University, MIE-CBI, CNRS UMR 8231, 10, Rue Vauquelin, 75231 Paris Cedex 05, France; 2 Chimie du Solide et de l’Energie, Collège de France, UMR 8260, 75231 Paris Cedex 05, France; 3 Sorbonne Université, Paris, France; 4 Réseau sur le Stockage Electrochimique de l‘Energie (RS2E), CNRS FR 3459, 75005 80039 Cedex, Amiens, France; 5 EDF R&D, EDF Lab Renardières, Département LME, 7 avenue des Renardières, 77818 Moret-sur-Loing cedex, France

**Keywords:** Energy storage, Batteries, Energy science and technology

## Abstract

In this study, the effect of flow of the electrolyte on an electrolysis cell and a zinc cell is investigated. The gain of energy brought by the flow is discussed and compared to the viscous losses in the cells. We point out that the balance between the gained electrical power and the viscous loss power is positive only if the hydrodynamic resistance of the circuit is correctly designed and further comment on the economical viability of the whole process. A model of the studied phenomena is proposed in the last section. This analytical model captures the dynamics of the process, gives the optimal flowing conditions and the limits of the energetical rentability of the process. This study shows that the use of flowing electrolyte in zinc–air batteries can be energetically profitable with the appropriate flowing conditions.

## Introduction

Oxygen electrochemistry has for many decades attracted a lot of attention in the scientific community because of its potential in the development of alternative energy technologies such as water electrolysers for the hydrogen production^1^, fuel cells or metal air batteries^2,3^. All these areas are currently active research areas because they are part of the fight against global warming. In all these technologies it is necessary to improve the efficiency of electrochemical reactions. Water oxidation or the oxygen evolution reaction (OER) is the core reaction for these processes along with oxygen reduction reaction (ORR) and/or the hydrogen evolution reaction (HER)^4^.

The equilibrium half-cell potentials ($E^0$) at 1 atm and 25$^0$C for OER and ORR are shown as follows in alkaline conditions:

1 $$\begin{aligned} 4 OH^- \rightleftarrows 2 H_2O(liq)+O_2(g)+4 e^{-} \end{aligned}$$

For pH = 14, $E^0 = 1.229 - (14*0.059)$ V = 0.403 V vs RHE ;

$E^0 = 0.404 - 0.197 V = 0.206$ V vs Ag/AgCl (sat KCl).

During OER , molecular oxygen is produced via several proton/electron- coupled steps. The reaction is highly pH dependent. In acidic and neutral conditions two water molecules ($H_2O$) are oxidized into four protons (H+) and one oxygen molecule ($O_2$), while hydroxyl groups (OH-) are oxidized to $H_2O$ and $O_2$ in basic environments^4^. The production of the oxygen molecule requires the transfer of four electrons. This proceeds through by a multi-step, multi-electron transfer, which have slow kinetic properties. This induces high overpotentials and low energy efficiency in practical devices. To circumvent this problem, it is necessary to develop catalysts^5^. The most promising catalysts at present are 3d transition metal oxides in alkaline conditions, and Ir-based oxides in acidic conditions. The electrolysis of water also encompasses the Hydrogen Evolution Reaction (HER). The equilibrium half-cell potentials at 1 atm and 25 °C for HER is shown as follows in alkaline conditions:

2 $$\begin{aligned} 2 H_2O(liq) +2 e^{-} \rightleftarrows 2 OH^- +H_2(g) \end{aligned}$$

$\text {E} = -1.023$ V vs Ag/AgCl(sat KCl) for pH=14.

Fast compared to the OER reaction in alkaline solution, the HER kinetics are drastically affected by a change of pH from acidic to alkaline. At pH 13, the HER activity is 2 or 3 orders of magnitude lower than at pH 0 for the same surface^6^. As for the OER, the penetration of water electrolysis in the hydrogen market production requires cheap and earth-abundant catalysts for the hydrogen evolution reaction. Platinum is still one of the best catalysts today^7^.

The slow kinetics of the OER or of the HER are not solely responsible for the low efficiency of the process. Overpotentials are also related to the ionic resistance of the electrolytes/membranes and the presence of bubbles on the electrodes^8^. Let us underline that the role of bubbles is complex. On the one hand, bubbles make the process less efficient. Attached to the electrodes, they mask them, decrease the effective surface area between the electrolyte and the electrodes, which creates an overvoltage. Present in the electrolyte, they decrease its conductivity and increase the ionic resistance. On the other hand, the presence of bubbles limits the amount of dissolved gaz and thus favorize the reaction. Bubble growth and even more bubble detachment, introduce convective flows that homogeneize the ionic concentration close to the electrode and avoid overpotential due to ionic concentration gradients^9^.

In the situation of high ionic concentration where the overpotential due to ionic gradients is low, in the situation of high intensity current where bubbles are numerous, make the bubbles leave the electrode is an efficient way to enhance the efficiency of the electrochemical processes.

The use of superhydrophobic surfaces or of external fields such as super gravity, ultrasound or magnetic fields have been proposed to prevent the bubbles from sticking to the electrodes^10,11^. In commercial electrolysers this phenomenon is partly combated by the use of electrolyte flow across the conductive substrate onto which the catalyst is loaded (required in a continuous process) which seems to enable the evacuation of bubbles^12^.

Zeng and Zhang^13^ obtained a small and in some situation no reduction in the cell voltage of an electrolyser when flowing electrolyte is used. Their studies focus on very large cells. In this situation the shear stress is low and unable to sweep efficiently the bubbles^12^. On the other hand, Phillips^12^, Bongenaar-Schlenter et al.^14^, Hine et al.^15^ showed that the electrolyte flow rate reduces the cell resistance in a finite gap cell, with large reductions in cell voltage even at high current densities (> 500 mA cm^−2^). Wang and coworkers^16^ showed that the overpotential of the gas electrodes in a zinc air battery decreases in presence of flow. They point out using numerical simulations and experiments that flow may avoid the coalescence of bubbles at the interface and promote the efficiency of the battery. This study is one of the few studies dealing with batteries and not electrolysers.

In this work, we will focus on these latter situations. Our goal is to understand whether introducing a flow that flushes out bubbles is an energetically efficient process. The cost and energy to construct and drive the flow has to be taken into account to ensure the economic profitability of the processes. Such studies have not been performed at this stage. In particular, the comparison between the viscous losses and the energy gains that are essential to understand whether the process is energy-effective or not have not been addressed.

**Figure 1:**
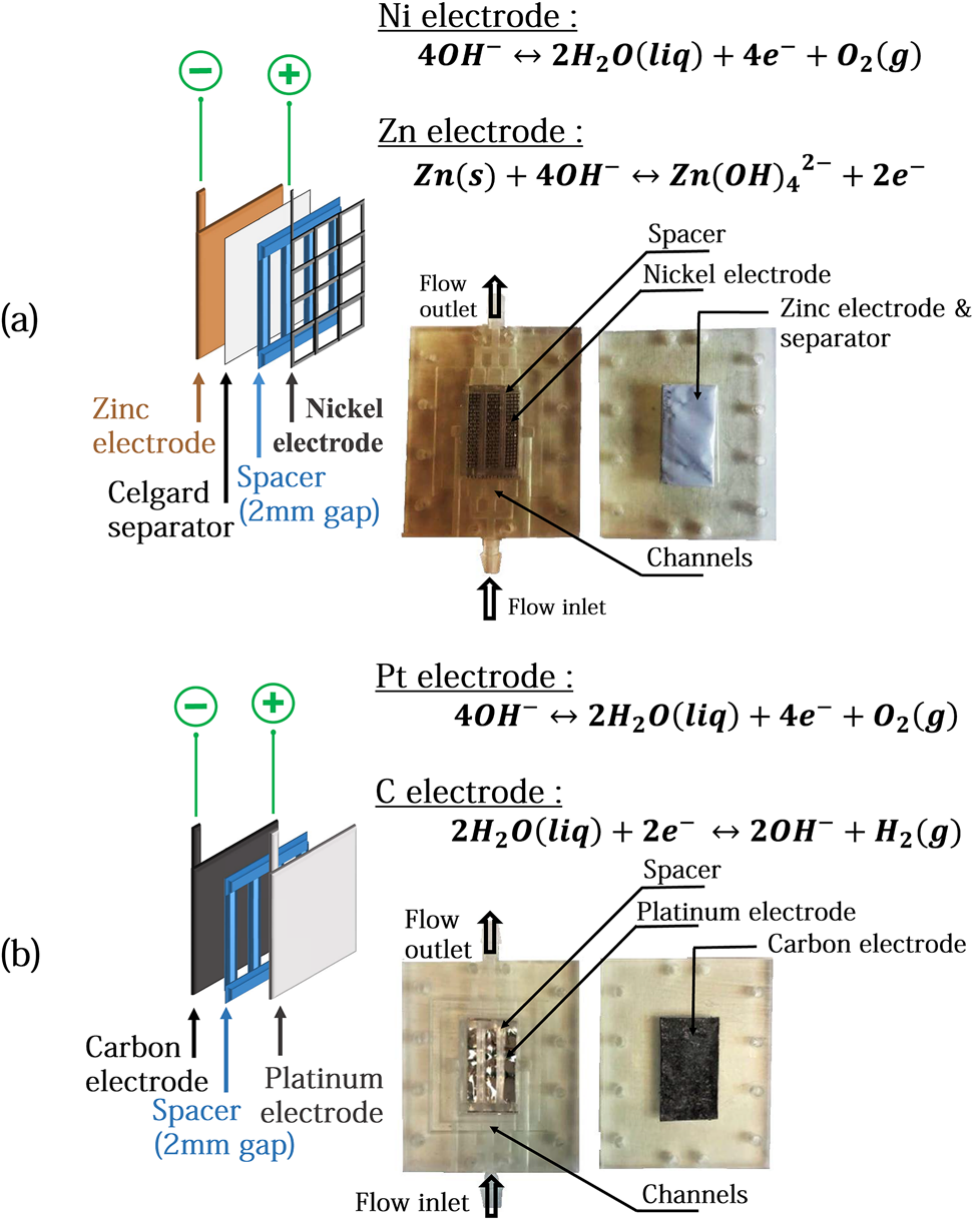
Sketches of the two systems studied. (**a**) Zinc Cell and its components. (**b**) Electrolysis Cell and its components.

For this purpose, we use two independent model systems:

An electrolyser made with a carbon and a platinum electrode respectively for HER and OER. The second system studied is a metal–air half-cell made with a zinc electrode and a nickel OER electrode. Zinc–Air complete batteries are often used in a configuration in which the Zinc electrode is connected to a gas-diffusion electrode during discharge and then switch to a gas-evolution electrode during the charge process^17,18^. In this configuration, the charge process is done on a separate electrode (usually a Nickel OER electrode) without damaging the fragile gas-diffusion electrode used in discharge for ORR. The cell used in this study can thus carry out the electrochemical process of charging zinc–air batteries. We study in detail the dynamics of production of gas bubbles during charge at the electrodes as a function of the current density. We write a model taking into account the variation of the electrodes active surface that captures this process. We numerically simulate the viscous losses and compare these simulations to in situ measurements. We explain under which conditions flowing electrolyte is an appropriate solution to improve the efficiency of the battery. Figure 1 illustrates the two systems and their different different components.

## Results

### Voltammetry experiments

The Voltammetry data are displayed in the supplementary material (see Fig S1, S2, S3, S4). At the platinum electrode, only the OER and the HER are visible on the voltammogram. The oxidation reaction appears to start at 0.6 V vs (Ag/AgCl). For higher potentials the current density follows the Tafel equation $I=I _0exp^{\frac{\alpha n F (E-E_{eq})}{RT}}$. We find $I_0=1.89$ $10^{-5}$ A and $\frac{\alpha nF}{RT}$=20.9. On the cyclic voltammetry of nickel, we clearly see additional oxidation and reduction peaks. After a few cycles, we observe for the positive currents a first peak around 0.5V vs Ag/AgCl. This peak is characteristic of the change in oxidation state from Ni(II) to Ni(III), where $\beta$-Ni(OH)$_2$ is oxidized to $\beta$-NiOOH. The electrochemical equation is:

3 $$\begin{aligned} \beta -Ni(OH)_2 +OH^{-} \rightarrow \beta -NiOOH + H_2O + e^{-} \end{aligned}$$

The oxidation current increase at a potential of approximately 0.6V vs Ag/AgCl, is attributed to oxygen evolution. For higher potentials the current density follows the Tafel equation (see supplementary materials Figure S4.). We find $I_0=7.2$ $10^{-3}$ A and $\frac{\alpha nF}{RT} = 13.9$. These parameters characterise kinetics of the oxygen evolution reaction at our Pt and Ni electrodes.

The electrochemistry of the carbon electrode in alkaline solution is more complex and leads to the formation of a variety of functional groups on the surface of the graphite^19,20^. This oxidation also leads to the degradation of the electrodes by the loss of graphitic surface layers when the layer becomes hydrophilic by the addition of oxygen functional groups. At negative currents these functional groups are reduced before the HER is observed. We find from the Tafel plot that $I_0=1.25$ $10^{-3}$ A and $\frac{-\alpha nF}{RT} = 12.4$. These parameters characterise the hydrogen evolution reaction at our carbon electrode.

### Cells voltage as a function of the flow rate

**Figure 2:**
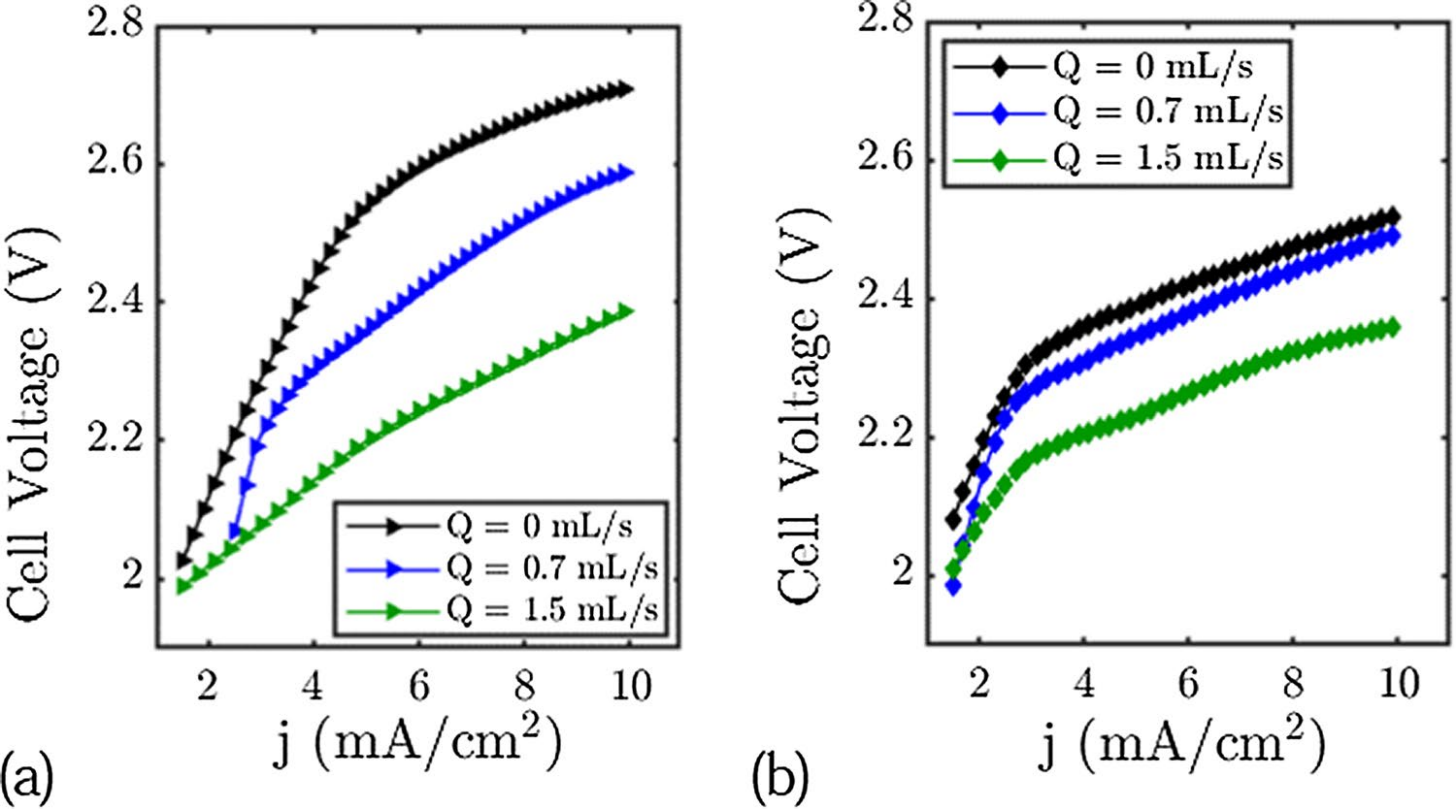
Cell Voltage of the electrolysis cell and the Zinc cell setup for different flow rates. (**a**) Electrolysis cell. (**b**) Zn-cell setup.

Figure 2 shows the cell voltage (the difference between the positive electrode and the negative electrode potentials) as a function of the current for different flow rates in the zinc cell and in the water electrolyser cell configuration.

In both cases we observe a decrease of the cell voltage of more than 150 mV for current densities higher than 5 mA cm^−2^ and flow rates higher than 1.5 mL/s (160 mV for the Zinc-cell and 340 mV for the Platinum/Carbon cell for a flow rate of 1.5 mL s^−1^). For current densities lower than 2 mA cm^−2^, the flow just has little effect on the cell voltage. We note that the decrease of the cell voltage is more important in the electrolysis cell. We anticipate that this is due to the electrolysis cell having two gas evolution electrodes. These data show the role of flow on cell voltage and confirm that the use of electrolyte flow is a good means of improving the performance of a rechargeable zinc cell or an electrolysis cell. In the next section, we will analyse what happens at each electrode.

### Chronopotentiometry experiments: zinc cell OER nickel electrodes

#### The zinc electrode

For current densities between j = 1 mA cm^−2^ and j = 10 mA cm^−2^, we observe little impact of the flow on the Zinc electrode overpotential (see Fig S5 in supplementary material). The variations of the cell voltage come from the variation of the Nickel OER electrode potential. Note that no gases are produced at the zinc electrode whereas oxygen is produced at the nickel electrode.

#### The nickel OER electrode

**Figure 3:**
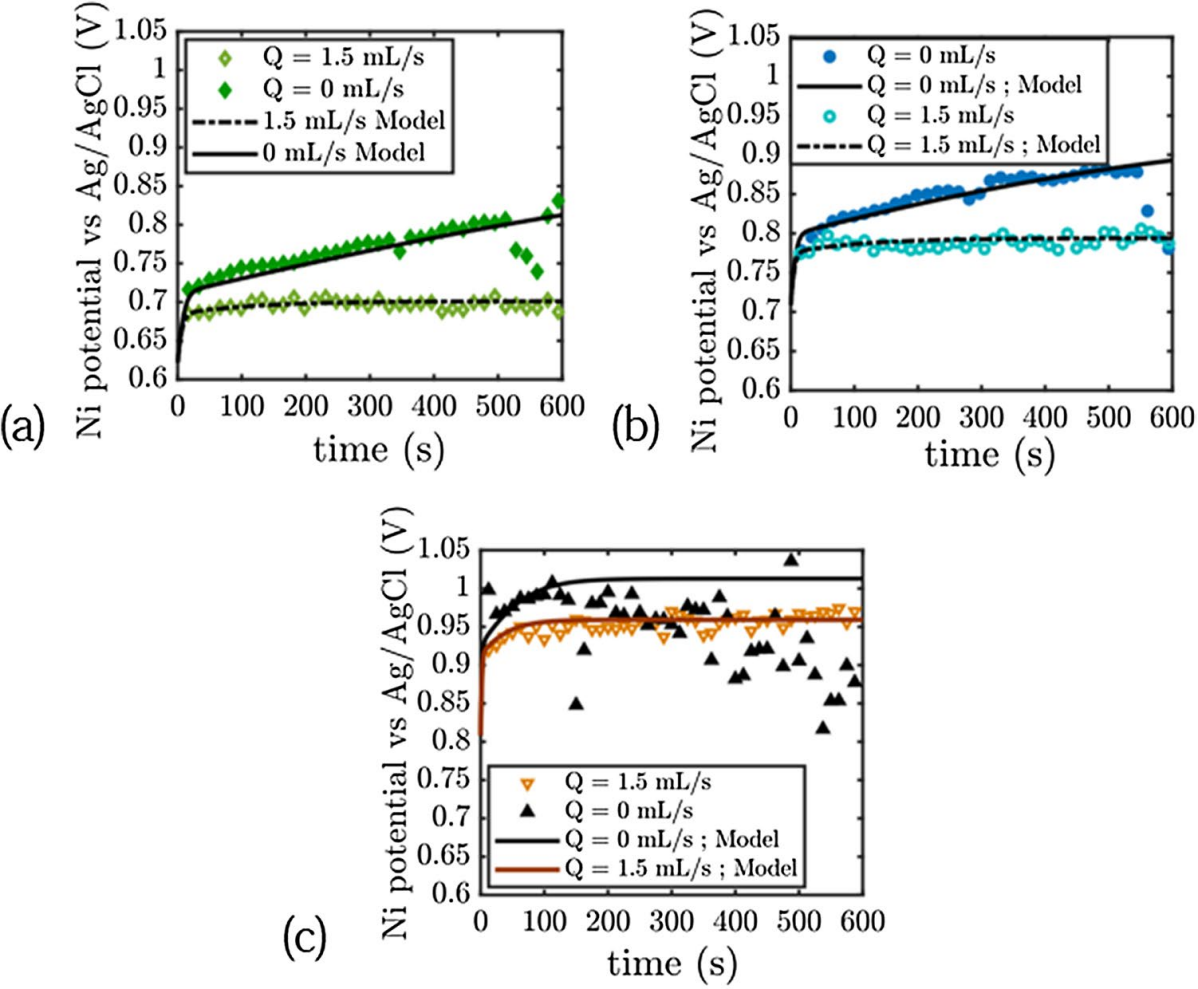
Chronopotentiometry of the Nickel electrode versus Ag/AgCl electrode during OER in 8M KOH/1M zincate electrolyte. (**a**) j  = 3 mA/cm$^{2}$. (**b**) j = 5 mA/cm$^{2}$. (**c**) j = 20 mA/cm$^{2}$.

Figure 3 displays what happens at the nickel electrode. The situation is very different to the zinc electrode. For this electrode where oxygen bubbles are generated, the electrode potential during charging is lower under flow. The electrode potential decreases as a function of the flow rate, for flow rate comprised between 0.3 mL/s and 1.5 mL s^−1^ and reaches a limit behavior for flow rate higher than 1.5 mL s^−1^. The overpotential of the electrode is continuously increasing in absence of electrolyte flow, and a plateau is reached after only a few seconds when the electrolyte is flowing. The difference in the potential of the electrode using static and flowing electrolyte varies between 100 and 150 mV and is due to bubble removal as expected. The process may not remove all the gas bubbles from the surface in this case, the electrode overpotential obtained solely from the Butler–Volmer equation (described in the voltammetry part) is lower than the measured ones: in the low current density region (j = 3 mA cm^−2^), the potential with flow decreases until 0.7 V versus Ag/AgCl, the theoretical voltage of the electrode according to Butler–Volmer equation and without taking bubble coverage in account at this current density is 0.67 V vs Ag/AgCl. The overpotential due to residual bubbles is thus limited and represents 30 mV for a flow rate of 1.5 mL s^−1^, while the value measured represents 130 mV for static electrolyte after 10 min of experiment. (We measure a value of 150 mV at steady-state for static electrolyte).

Increasing the current density leads to an increase of this overpotential: removing all the gas bubbles from the electrode surface at j = 20 mA cm^−2^ should give a potential around 0.8 V vs Ag/AgCl using the Butler–Volmer equation while 0.95 V vs Ag/AgCl is measured with flowing electrolyte and more than 1 V with static electrolyte. In the high current density situation, we also measure noisy signals in the absence of flow and smooth signals when there is flow. These noisy signals (150 mV amplitude at j = 20 mA cm^−2^ in Fig. 3) can be attributed to the departure of large bubbles from the electrodes. The large potential fluctuations appear after a certain time, which is inversely proportional to the current density. When this time has elapsed, the fluctuations appear randomly (typically after 130 s in Fig. 3). We will come back to the explanation of this phenomenon in the modeling section.

### Chronopotentiometry experiments: platinum/carbon electrolyser

#### The platinum electrode

**Figure 4:**
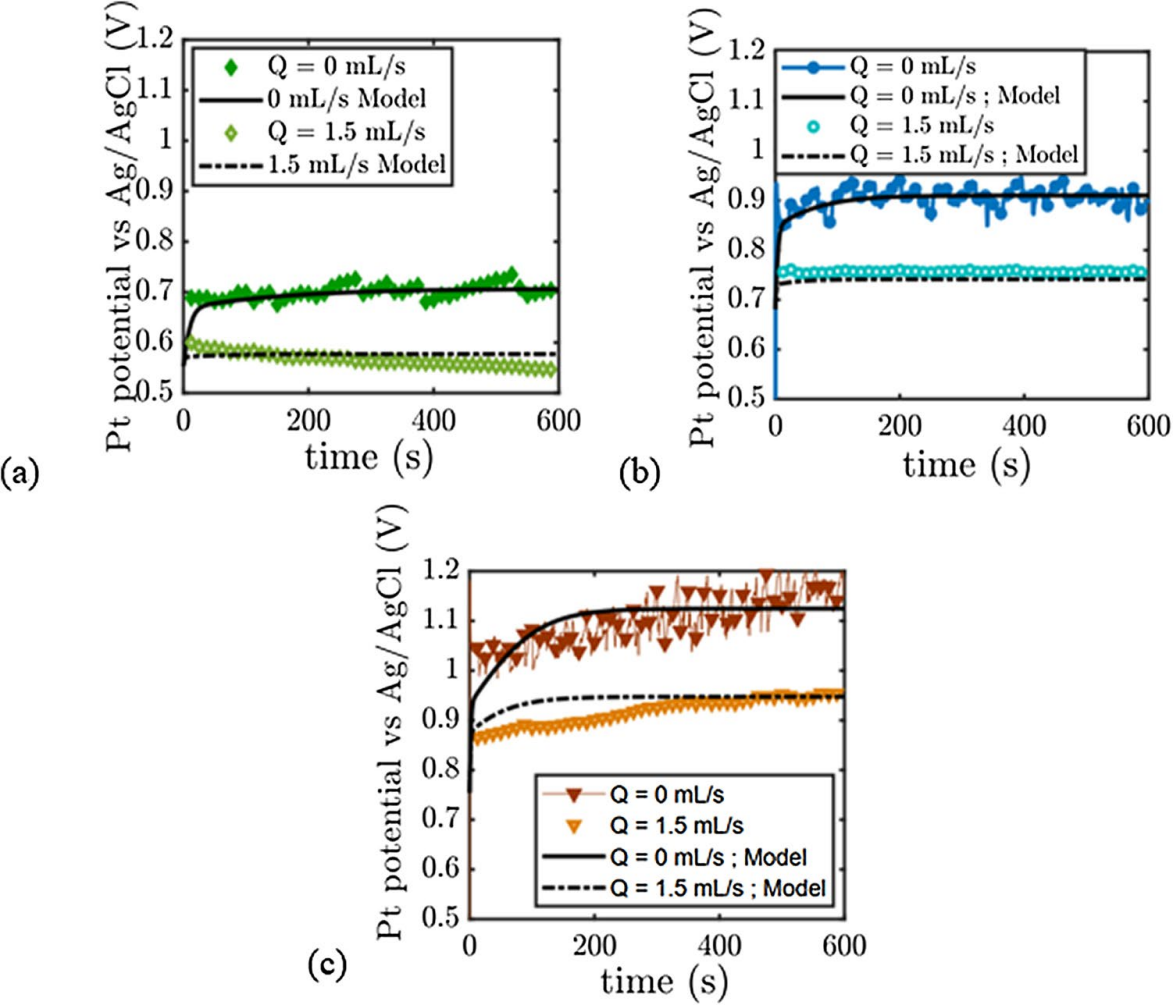
Chronopotentiometry of the Platinum electrode versus Ag/AgCl electrode during OER in 8M KOH/1M zincate electrolyte. (**a**) j = 3 mA/cm$^{2}$. (**b**) j = 10 mA/cm$^{2}$. (**c**) j = 20 mA/cm$^{2}$.

Figure 4 displays what happens at the Platinum plate.As in the previous section, the overpotential at the Pt electrode during oxygen production is reduced with flow. For j = 3 mA cm^−2^, we measure a potential of 0.55 V (0.7 V for static electrolyte), and the calculated overpotential from the Butler–Volmer equation gives approximately the same value. All the oxygen bubbles appear to be evacuated by the flow. Increasing the current density decreases the efficiency of the flow process as seen in the previous subsection: for j = 20 mA/cm$^{2}$, bubble-free surface of the electrode should give 0.75 V (vs Ag/AgCl) while we are measuring 0.95 V. The process still has its interest because the potential with static electrolyte at this current density is around 1.15 V. We note that the fluctuations in potential in the absence of flow at high current densities (typically less than 0.1V at j = 20 mA/cm$^{2}$ in Fig. 4) are of smaller amplitudes than at the Nickel grid. This can be due to differences in the nature of the electrode, in geometry, and surface differences which modifies the dynamics of bubbles departure from the electrode. At low current density, we also see that the measurements decrease slowly towards a plateau value which varies between 0.6 and 0.55 Volt. We will comment this point in the modeling section.

#### The carbon electrode

**Figure 5:**
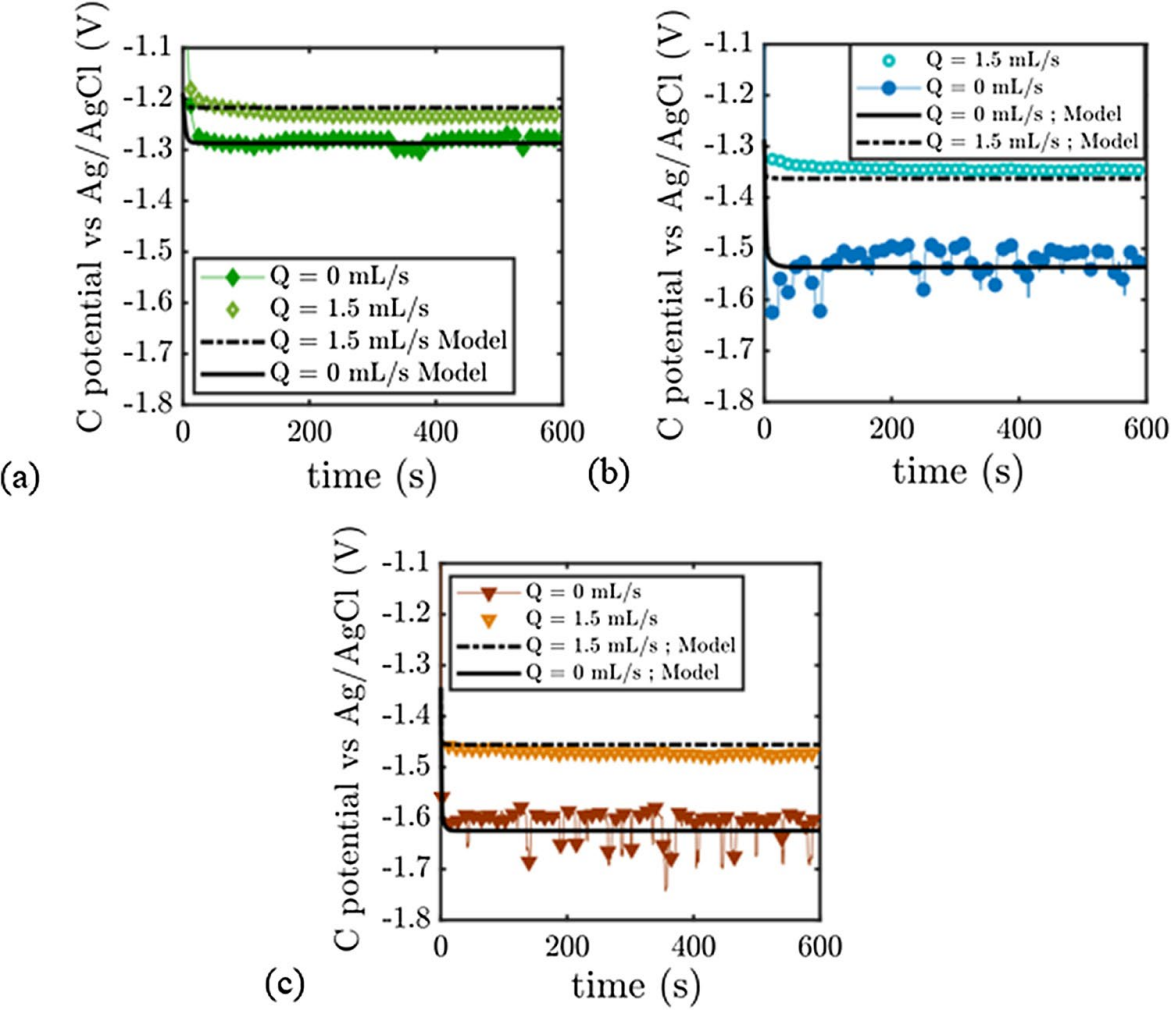
Chronopotentiometry of the carbon electrode versus Ag/AgCl electrode during OER in 8M KOH/1M zincate electrolyte. (**a**) j = −3 mA/cm$^{2}$. (**b**) j = − 10 mA/cm$^{2}$. (**c**) j = − 20 mA/cm$^{2}$.

Figure 5 displays what happens at the carbon plate during HER. The electrode exhibits a similar behaviour to those observed above.

The electrode potential during hydrogen production increases with flow: in the low current density situation, we measure − 1.23 V vs Ag/AgCl with flowing electrolyte, and − 1.29V vs Ag/AgCl with static KOH; the theoretical potential for a bubble-free electrode/electrolyte interface (pH14) is − 1.20 V vs Ag/AgCl. As seen previously the process is less efficient at high current density: For j = 20 mA cm^−2^, the calculated potential of the bubble-free electrode by the model is − 1.34 V vs Ag/AgCl, whereas it is measured to be − 1.47 V with flowing electrolyte (Q = 1.5 mL s^−1^), and around − 1.66 V at Q = 0 mL s^−1^. We observe a large variation of the potential under flow which is close to 200 mV at j = 20 mA cm^−2^ in Fig. 5. In the high current density situation, the amplitudes of the potential fluctuations with static electrolyte are close to 100 mV.

### Bubble visualisation

In order to link the measured signals and the mechanisms of bubble formation and detachment, we performed optical measurement using the device described in the experimental section. These measurements can be made more simply on the nickel grid and would be more complex on the Platinum and Carbon plates which are solid and opaque. We will present detailed measurements on the Nickel grid and more scattered measurements on the other two electrodes. The influence of current density and flow rate on bubbles formation and detachment are investigated.

**Figure 6:**
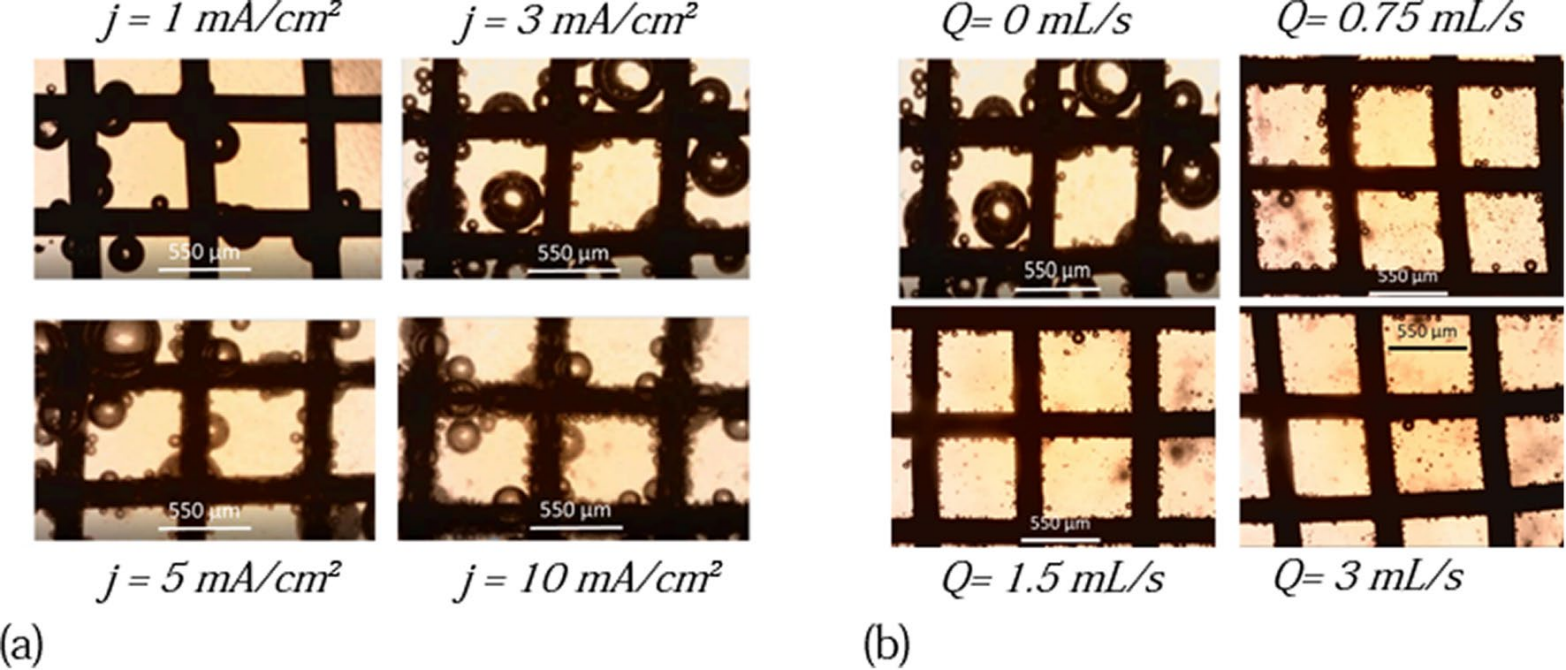
Picture of the bubbles on a grid of Nickel (**a**) as a function of the intensity current for Q = 0 mL s^−1^. (**b**) As function of the flow rates for j = 3 mA cm^−2^.

On the Nickel grid, (see Fig. 6a) for current densities below 1 mA/cm$^2$, we observe the formation of bubbles that grow very slowly. Typically 2–3 bubbles appear simultaneously on the same mesh.These bubbles grow, meet and coalesce to occupy the entire mesh (the side length of a mesh is 550 microns). When two bubbles occupying two different meshes meet and coalesce, the resulting bubble leaves the electrode under the action of Archimedes’ buoyant force. The surface tension forces are no longer large enough to hold it on the grid. The average value of the radius of the bubbles leaving the grid is 550 microns. This radius might correspond to the Fritz radius i.e the size at which the buoyancy force equals the adhesion. Note that this radius might be smaller^21^. Indeed the excess of surface energy generated during coalescence might be enough to overcome the adhesion energy. After a large bubble leaves, new bubbles form. These bubbles appear at the same nucleation sites as the previous ones which suggests that those are preferred nucleation spots. At these currents,the number of bubbles remains very limited.

For current densities of the order of 1 mA cm^−2^, a second population of bubbles of smaller radius appears. These small bubbles leave the electrode quickly in spite of their size (typically around 30 microns) and do not have time to grow. This means that they are formed at different sites from the first ones, they are less retained by the grid. This may be due to surface differences. The grid displays inhomogeneities, possibly also in the thickness of the nickel hydroxide surface layer. At these currents, the small bubbles have no impact on the large ones which follow the same evolution as for lower currents. For current densities above 3 mA cm^−2^, the number of small bubbles increases. Their departure from the grid and the hydrodynamic recirculations that accompanies it favors the release of the large bubbles. The radius of the large bubbles that detach decreases from 550 micrometers for 1 mA cm^−2^ to 100 micrometers for 3 mA cm^−2^ which is certainly below the Fritz radius.

**Figure 7:**
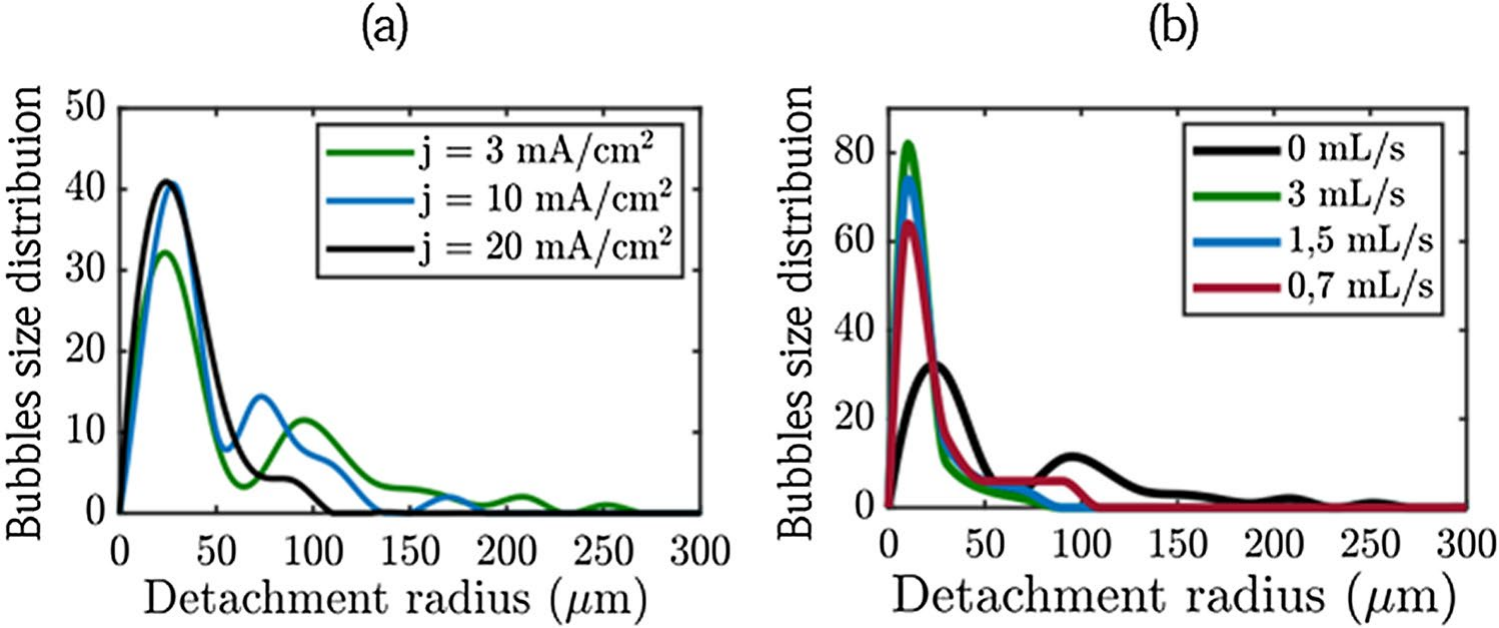
Size distribution of bubbles before departure from the Ni grid. (**a**) Influence of the current density at flow rate Q = 0 mL/s. (**b**) Influence of the flow rate at current denstity j = 3 mA cm^−2^.

When they leave the electrodes, the small bubbles form a plume^22^. They are separated by a given distance and leave perpendicularly from the interface. By increasing the current density from 5 to 20 mA cm^−2^ the production of small bubbles becomes more and more intense, decreasing the critical radius of the large bubbles. At 20 mA cm^−2^, the average of the critical radius of large bubbles reaches 74.55 microns, their lifetime on the grid is low, their growth is mainly due to coalescence events. The same behavior (i.e the existence of a bimodal population) is observed on the platinum plate in the electrolysis cell. However we do not observe bubbles reaching 550 microns (ie the size of the mesh of the Nickel grid) at low current densities because the geometry of the electrode is different. Figure 7a displays the bubble size distribution on the Ni grid for three current densities. These data were obtained by statistical analysis over 100 bubbles for each current density. The bubble distributions confirm the previous descriptions. The distribution is bimodal as reported in previous experiments on water electrolysis^22^: in the first 10 min after initiating the current, the bubble distribution is bimodal. It evolves toward a log normal distribution when steady state is reached, which takes more than two hours. Note that experiments performed on an electrode with designed cavities gives a single mode distributrion^23^. The electrodes surfaces are non-ideal and can present inhomogeneities coming from their natural roughness. Gas cavities on non-ideal surfaces are known to be nucleation and growth sites for bubbles. The electrodes used in this study can present different type of gas cavities leading to different population of bubbles. In the case of the Nickel electrode, the hydroxide layer formed during oxidation can generate other cavities reinforcing the surface inhomogeneity of the electrode.

The number of small bubbles increases with the current and then plateaus. The average size of the large bubbles decreases with the current. Due to the opacity of the platinum plate and the carbon plate, the transmission microscope could not be used to measure and acquire the same data. However, the following conclusions can be drawn. In all cases the distribution is bimodal. The small bubbles on the platinum plate have an average size of 30 microns, the radius of the large bubbles decreases with the current from 122 to 70 microns. On the carbon electrode, the hydrogen bubbles are much smaller, the smallest ones have a radius of 15 microns and the largest ones have a radius of 45 microns which does not vary too much with the current density.

We also used this cell to perform experiments under flow. Figure 6b shows pictures of the nickel grid under a current density of 3 mA cm^−2^ for several flow rates at steady-state. The number of bubbles from both population on the electrode decreases significantly when the flow rate is increased. This behavior is more pronounced for large bubbles (see Fig. 7b). For high flow rates, the distribution tends to a uni-modal distribution. Increasing the value of the flow rate also decreases the detachment radius of the two populations.

## Discussion

In the following section we will propose a model of the observed phenomena based on a phenomenological approach. In this simple model we describe the effect of the flow on the bubble removal from the electrode. In this model different parameters are fitted. We analyze these fitting parameters and compare them to experimental measurements.

### Reduction in energy consumed

**Figure 8:**
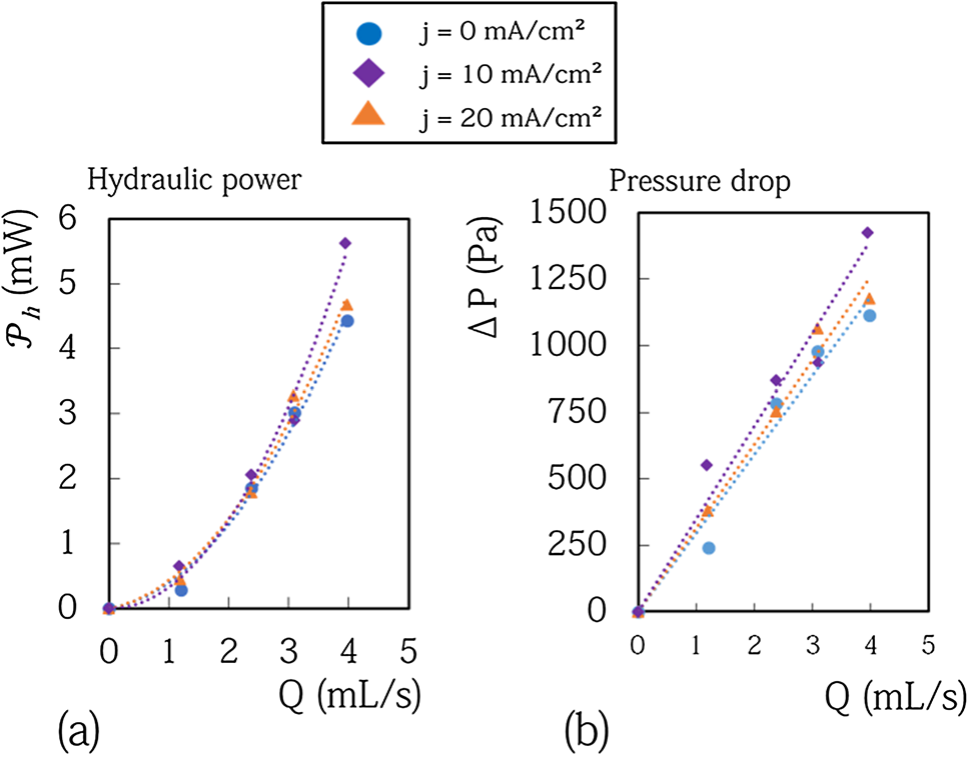
(**a**) Hydraulic power required to flow the electrolyte in the cell for various flow rate and various current density. (**b**) Drop of pressure as a function of the flow rate for various current density.

The previous measurements show a significant reduction in the overpotential of the bubble generating electrodes with flowing electrolyte. The performances of the cells are therefore improved : the cells require less power in operation. A zinc cell can thus be charged faster for a fixed voltage and can be charged with higher energy efficiency for a given charge current. From an economical point of view, a reduction between 150 and 300 mV in these configurations is a real gain. It corresponds to a gain around 10% on the overall power of the cells. To corroborate this, it is necessary to compare the power gain due to the reduction in overpotential with the power required to circulate the electrolyte in the cell $$\mathcal {P}_{h}$$. We have measured the pressure drop in the cell $$\Delta P$$ as a function of the flow rate and deduced the power of the viscous losses $$\mathcal {P}_{cell=\Delta P Q}$$. The data are displayed in Fig. 8. The measured power dissipated in the cell due to viscous loss is less than 6 mW whatever the experimental conditions. These values are in perfect agreement with a crude estimate of the viscous loss that can be made assuming low Reynolds number (less than 100) and neglecting the drop of pressure in the corners and in the junctions. In this situation the power dissipated for rectangular sections writes $$\mathcal {P}_{h} =\frac{12Q^2\eta L_{cell} }{h^3_{cell} w_{cell}}+6 \frac{12Q^2\eta L_{channel}}{h^3_{channel} w_{channel}}$$ where Q is the flow rate, $$ h_{cell}$$the thickness of the cell which is equal the thickness of the spacer, $$h_{channel}$$ the thickness of the channel, $$L_{cell}$$ the length of the cell, $$L_{channel}$$ the length of the channels, $$w_{channel}$$ the width of the channel, $$ w_{cell}$$ the width of the cell. The prefactor 6 for the channels term comes from the number of channels present in the cell. For our cell we have: $$\frac{12\eta L_{cell} }{h^3_{cell} w_{cell}}$$=1.17 10$$^6$$ Pa s m$$^{-3}$$. For the 6 channels the calculation gives: $$6 \frac{12 \eta L_{channel}}{h^3_{channel} w_{channel}}$$=1.37 10$$^8$$ Pa s m$$^{-3}$$. This shows that the viscous losses happen mainly in the channels. This analysis leads to an estimated dissipated power equal to 2.8 mW for Q = 4 mL s^−1^. This value is in good agreement with the measurement equal to 4 mW for Q = 4 mL s^−1^. The disagreement between the estimated values and the measured ones could be related to the fact that the previous expressions are formally valid only for $$\frac{w}{h} > 10$$, which is not the case for the channels. To conclude let us highlight that the measurements are very little affected by the presence of electric current. This means that the bubbles present in the electrolyte only slightly modify its viscosity.

According to the previous measurements, the power required to circulate the electrolyte in the whole circuit is equal to 0.3 mW for a flow rate of Q = 1.5 mL s^−1^. For the Zinc-cell, the viscous losses for Q = 1.5 mL s^−1^ corresponds to 2.9% of the power gained due to the decrease of the overpotential $P_{elec}=I\Delta V$ where dV is the overpotential and I the current ($P_{elec}$=13 mW). The net power gain at j = 10 mA/cm$^{2}$ is 12.7 mW and represents 6$\%$ of the overall power of the cell. For the electrolysis cell, viscous losses represent 1.6$\%$ of the power gained and the net power gain at j = 10 mA cm^−2^ is 21.13 mW which represents 10$\%$ of the overall power of the cell.

At this stage, the dissipation in the tubing have not been discussed. In our set up they are negligible. They write $\mathcal {P}_{tubing}=\frac{8Q^2\eta L }{R_t^4}$, and corresponds to 185 $\upmu$W for Q = 5 mL s^−1^ with $R_t$ the radius of the tubing (here 3mm), $L_t$ the tubing length (here 30 cm). These losses vary as a function of $R_t$ at the power 4 suggesting that the use of tubing with a radius of 1 mm instead of 3 mm will lead to an increase of the dissipated power in the tubing of 81 times. This shows clearly that a particular care in the choice of the tubing has to be brought in order to get a positive balance at the end.

### Modeling

The hypothesis used is that the decrease in the overpotential is related to the release of the bubbles by the electrolyte flow. Sweeping the bubbles away increases the effective or working area of the electrodes and thus decreases the overpotential. We have taken into consideration two bubble populations.This hypothesis is based on our observations and on previous reported experiments^22^. The originality of our model is to capture the temporal evolution of the overpotential which was not the case in the previous models^24,25^.For the sake of simplicity we assume the following hypotheses: the size of the bubbles for each population is constant. The size of the small bubbles is set to $R_b=15\,\upmu$m. The size of the large bubbles $R_a$ varies as a function of the flow rate and of the current density. We define $N_a(t)$ and $N_b(t)$ as the number of bubbles of respectively size $R_a$ and $R_b$ that remain on the surface. We assume that bubbles appear at a given radius on the surface $R_a$ or $R_b$ and that they leave the surface after a given characteristic time. Coalescence or Ostwald rippening are not considered.

The volume of oxygen produced, and indirectly the number of bubbles, follows the Faradaic process and is therefore proportional to the current; we also assume 100% coulombic efficiency for the OER. In the following, we define I as the current flowing across the electrode. The evolution of the number of bubbles on the surface is ruled by three processes: (1) the number of bubbles produced by the Faradaic process (2) the probability of bubbles departure and (3) the electrolyte flow. Bubbles are produced by Faradaic reaction. We assume that the number of bubbles produced by unit time $\alpha$ is only a function of the current density. We define $\alpha _a(I)$ as the number of bubbles of size $R_a$ and $\alpha _b(I)$ the number of bubbles of size $R_b$. The probability of departure of a bubble from the surface is assumed to be only a function of the current density. We define $-\beta _a(I)N_a$ as the number of bubbles that leaves the surface by unit time for the bubbles of size $R_a$ and $-\beta _b(I)N_b$ for bubbles of size $R_b$. For the sake of simplicity, we do not consider that the departure from the surface may be faster when the number of bubbles is higher. This would require the inclusion of terms depending on $N_a^2$ or $N_b^2$. This dependence will be included in the variation of the parameter $\beta$ and $\gamma$ as a function of the current. We assume that the flow increases the frequency of departure of the bubbles, and that this increase is proportional to the flow rate. We define $-\gamma _a(I)Q N_a$ as the number of bubbles that leaves the surface by unit time due to the flow for the bubbles of size $R_a$, and $-\gamma _b(I)QN_b$ for bubbles of size $R_b$. We take the assumption that the parameters $\alpha _a(I), \alpha _b(I) \beta _a(I) \beta _b (I) \gamma _a(I) \gamma _b(I)$ depend on I and not on the local current density *j*, a mean field approximation which is the basis of this simple model. As the surface decreases when bubbles are present, the local current density value increases with time during the chronopotentiometry experiments. We have neglected these second order variation in the model.

The conservation of the number of bubbles is expressed by:

4 $$\begin{aligned} \frac{dN_a(t)}{dt}= & {} \alpha _a(I)-\beta _a(I)N_a-\gamma _a(I) Q N_a \end{aligned}$$

5 $$\begin{aligned} \frac{dN_b(t)}{dt}= & {} \alpha _b(I)-\beta _b(I)N_b-\gamma _b(I)Q N_b \end{aligned}$$

The parameters $\alpha _a(I)$, $\alpha _b(I)$ are linked by the volume of oxygen produced.

6 $$\begin{aligned} \frac{ d V_{O_2}(t)}{dt}=\frac{V_m I }{4 F} =\alpha _a(I)V_a+\alpha _b(I)V_b \end{aligned}$$

$V_{O_2}(t)$ is the oxygen volume produced after time t, F is the Faraday constant, $V_m$ is the molar volume and $V_a$ & $V_b$ are the volumes of the bubbles of radius $R_a$ & $R_b$ respectively.

This set of equation enables us to calculate the active surface *S*(*t*)of the electrode as a function of time :

7 $$\begin{aligned} S(t)=S_o-N_a(t)S_a-N_b(t)S_b \end{aligned}$$

where $S_a$ and $S_b$ are the surfaces covered by bubbles from population a and b. The expression of these surfaces are related to the local supersaturation of gas, to the contact angle (which varies with the intensity of the electric field and which depends according to the material), and to the surface of the electrode^24,26^. In our situation, the electrolyte is wetting the electrodes. We assume here an average value for the covered surface by bubbles which is proportional to $\pi r_{a,b}^{2}$ by a factor K. Following^24,26,27^, we take K = 1. Such definition of the bubble coverage is correct because the current density on the shaded area below the bubbles is nil or very low. $S_0$ corresponds to the total surface of the electrode in contact with the electrolyte when there is no bubbles on its surface.

Finally, the Butler–Volmer equation allows us to find the evolution of the potential taking into account the surface evolution:

8 $$\begin{aligned} E=E_{eq}+ \frac{R T}{z\alpha _0F} ln(\frac{S_0}{S(t)}) + \frac{R T}{z\alpha _0F} ln(\frac{I}{I_{0}}) \end{aligned}$$

*I* is the current over the electrode. $I_{0}$ and $\alpha _0$ are deduced from the Tafel fit. These equations take into account the overpotential due to the kinetics of the reaction in absence of bubbles and the overpotential due to the presence of the bubbles coined hyperpolarization of the electrode in^28^. We have neglected the Ohmic loss due to the internal resistance of the cell, the overpotential created by the concentration gradients close to the interface, the overpotential created by the solubilization of gazes in the electrolyte. Each of these overpotential are comprised between 1 and 10 mV in our situation^28^.

During the Voltammetry experiments, the scan velocity is high enough to avoid the production of bubbles over the electrode since the charge passed through this short experiment remains quite low. Voltammetry experiments were also performed in presence of flow to determine $I_0$, in both cases we report the same values. This model has five independent fitting parameters $\alpha _b(I),\beta _a(I),\gamma _a(I),\beta _b(I),\gamma _b(I)$ and can be resumed by the equations 4, 5, 7, 8. Using a least squares procedure to fit the experimental values (over various voltammetry data for various flow rates), we calculated them as a function of the current. $R_a$ and $R_b$ have been measured in the experimental section and are not fitting parameters.

The chronopotentiometry experiments are a close fit to this simple model (see Figs. 3, 4, 5). Before analysing the measured parameters, let us return to the remark made in the chronopotentiometry section. The fit of the low current model is not as good as the high current model in terms of kinetics. The experimental points are above the model. This is due to the fact that at low current, the initial state of the surface is very important. If bubbles have not been expelled from the surface, these bubbles will take some time to be expelled and will result in higher potential than expected. At high currents, these bubbles are very quickly masked by the bubbles created by the current so that the fit works perfectly.

Table I, II and III display the values of these phenomenological parameters as a function of the current. The three electrodes tested share the same behavior. $\alpha _a(I)$ and $\alpha _b(I)$ are both proportional to *I*, they check Equation 6. We recall that $\alpha _a(I)$ is not a fitting parameter it is calculated from $\alpha _b(I)$. $\alpha _b(I)$ is higher than $\alpha _a(I)$ because the number of small bubbles is higher than the number of large ones. Also, the total volume of oxygen $\alpha _a V_a$ present in large bubbles is less than the total volume of oxygen $\alpha _b V_b$ in small bubbles even if the population A is larger in size. $\beta _b(I)$ is higher than $\beta _a(I)$. This is consistent with what is expected. All bubbles develop at the same rate, it naturally takes longer for a large bubble to reach the largest size.

**Figure 9:**
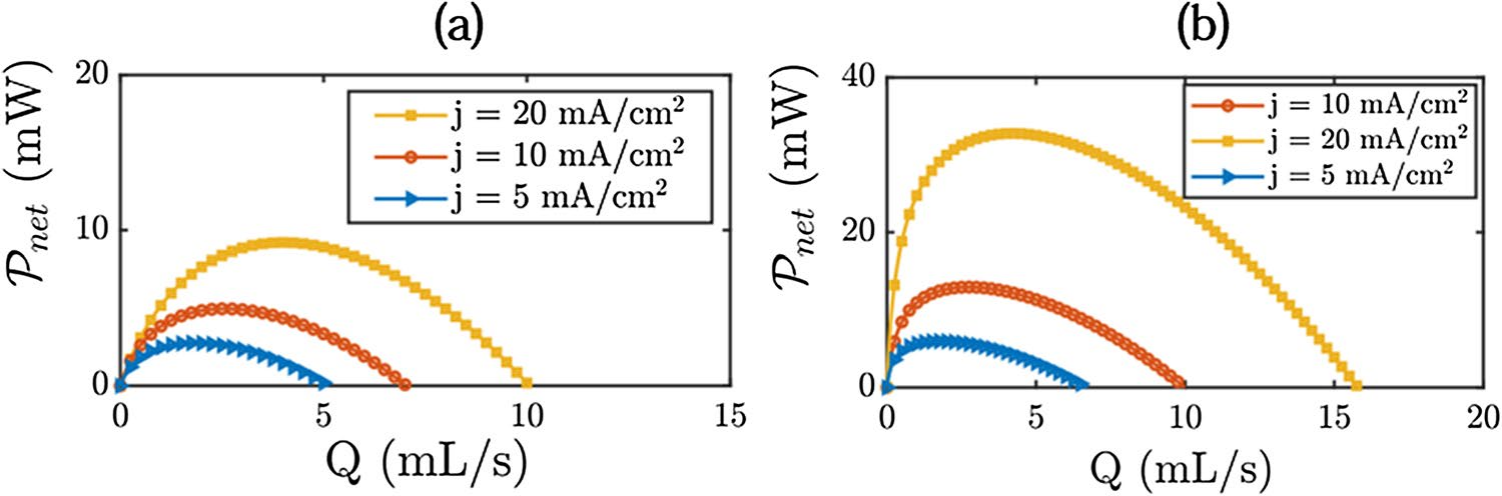
Net power gained calculated by the analytical model for the different systems studied. (**a**) Zinc cell. (**b**) Electrolysis cell.

We recall that $\gamma$ is the flow parameter which gives the number of bubbles released by the electrolyte flow per unit time. $\gamma _a$ values are lower than $\gamma _b$ ones, at first sight it is also surprising since the drag force of the liquid exerted on a bubble increases with the square of the bubble radius and should thus be higher for larger bubbles^24^. However, as shown by microscopic visualisation, small bubbles form trains of bubbles and the drag force in the plume is higher than the one close to a surface. This explains why the parameter $\gamma$ decreases as a function of the bubble size. $\gamma$ values do not depend upon the intensity of the current. This comes from the fact that the drag force is not related to the bubble formation frequency.

As mentioned at the beginning of the paragraph, the three electrodes tested have similar fit parameter values. However, there is an important difference in the $\gamma$ parameters. The effect of flow is more modest on a Nickel grid than on Carbon and Platinum plates. It could be due to the geometry of the electrode which can keep bubbles trapped in the mesh. The greater the $\gamma$ parameter, the greater the energy gain on the battery or electrolyser. A plate would therefore be more efficient for the application in this specific configuration.

The results in Fig. 3 illustrate this lower efficiency, especially at high current density. At j = 20 mA cm^−2^, the potential difference between flowing and static electrolyte is about 60 mV, against 150 to 200 mV for the carbon and platinum electrode. Linear interpolations of the numerical parameters evolution enables the potential of the electrode to be calculated for higher current densities at a given flow rate.

**Table 1:** Modelling parameters for the platinum electrode.

J (mA cm^−2^)	3	5	7.5	10	20
R$_a(\upmu$m)	120	90	79	77	70
R$_b$($\upmu$m)	15	15	15	15	15
$\alpha _a(I)$	7	20	23	31	69
$\alpha _b(I)$ 10$^4$	4	7	11	15	30
$\beta _a(I)$	0.0070	0.0085	0.0120	0.0180	0.0215
$\beta _b(I)$	0.14	0.2	0.31	0.4	0.78
$\gamma _a(I)$	6000	6000	6000	6000	6000
$\gamma _b(I)$ 10$^5$	3	3	3	3	3

**Table 2:** Modelling parameters for the nickel electrode.

J (mA cm^−2^)	3	5	7.5	10	20
R$_a(\upmu$m)	120	100	90	85	75
R$_b$($\upmu$m)	15	15	15	15	15
$\alpha _a(I)$	7	16	31	46	132
$\alpha _b(I)$ 10$^4$	7	13	19	26	52
$\beta _a(I)$	0.0023	0.0035	0.0067	0.0079	0.0248
$\beta _b(I)$	0.12	0.21	0.29	0.43	0.76
$\gamma _a(I)$	4000	4000	4000	4000	4000
$\gamma _b(I)$10$^4$	2.5	2.5	2.5	2.5	2.5

**Table 3:** Modelling parameters for the carbon electrode.

J (mA cm^−2^)	3	5	7.5	10	20
R$_a(\upmu$m)	45	45	45	45	45
R$_b$($\upmu$m)	10	10	10	10	10
$\alpha _a(I)$	85	119	153	171	339
$\alpha _b(I)$ 10$^4$	30	50	75	102	196
$\beta _a(I)$	0.0850	0.0931	0.1010	0.1150	0.2561
$\beta _b(I)$	0.40	0.54	0.76	1.01	1.98
$\gamma _a(I)$	8000	8000	8000	8000	8000
$\gamma _b(I)$ 10$^5$	4	4	4	4	4

Figure 9 shows the modelling results of the total power reduction in charge by using flowing electrolyte. These results take in account the viscous losses in the cells. Viscous losses increases with the flow rate and the modelling results allow to define the flowing conditions for a positive energy gain. This gain is positive for flow rates lower than 10 mL s^−1^ for the Zinc cell and lower than 15 mL s^−1^ for the electrolysis cell. The optimal flow rate corresponds to the maximum of the curves shown in Fig. 9. The power gained is higher on the electrolysis cell which presents two gas evolution electrodes.

### Conclusion

In this work, we have studied the behavior of gas-evolving electrodes in presence of electrolyte flow. We introduce new elements in favor of the use of flow batteries outside the renewable energies sector. It has previously been shown that flow may reduce the dendrite formation. We have shown that electrolyte flow also decreases the overpotential at the electrodes producing gas. This behavior is quite general and occurs both on a platinum plate and on a nickel grid electrode. The reason is simple: the flow sweeps the bubbles and avoid the decrease of the effective area of the electrode. We point out that this may induce 7.5% of gain on the overall charge power of the battery for a current density less than j = 20 mA cm^−2^. This gain takes into account the viscous losses induced by the circulation of the electrodes.

These losses can be easily decreased by using an appropriate hydraulic circuit which avoids high hydraulic resistance. We have also developed a simple model to predict the performance of the cell. We believe that such a model is important for the scale up and industrialization phases of a battery. As the flow process is cost-effective, it could be coupled with zinc air batteries for vehicles. It should be noted that fuel cells have already been proposed for this type of application^29,30^.

## Methods

### Electrolytes

The electrolyte used to study the effect of the flow on the electrodes potentials is an 8M aqueous solution of potassium hydroxide. This electrolyte is prepared by dissolving KOH (Sigma-Aldrich) in deionised water. The high concentration of hydroxide ions ensures the high conductivity of the solution. For the Zinc cell, zincates ions at a concentration of 1M are electrochemically added to the 8M KOH solution to ensure zinc electrodeposition at the Zn electrode and avoid HER during the charge process.

### Millifluidics cells for chronopotentiometry

**Figure 10:**
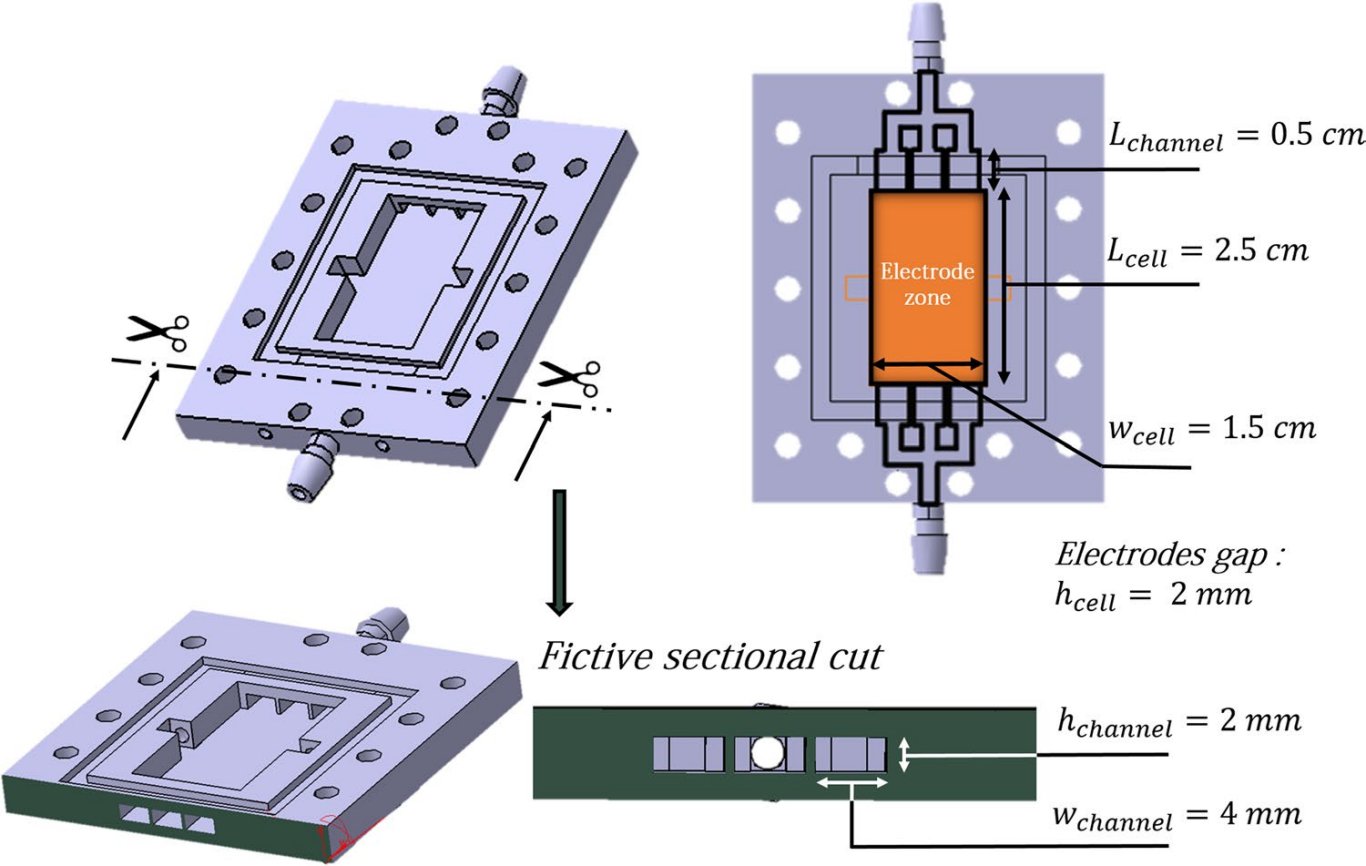
**CAD** sketch of the cell and associated dimensions. The output and input channels are connected to a Tank of electrolyte by a tubing (PharMed BPT, Saint-Gobain) with an internal radius of 3 mm and a length of 30 cm.

To test the various experimental conditions, two experimental home-made devices are used as a flow systems (see Fig. 10): a Zinc cell and a Platinum-Carbon electrolysis cell. Both cells are used vertically. In both situations, we used a 3D UV resin printer (Form 3, Formlabs) to create the network of channels necessary for electrolyte circulation. The resin used is a mixture of methacrylic acid esters and photoinitiator (Clear Photoreactive Resin for Formlabs 3D printers, Formlabs) and the resulting shapes are chemically stable towards hydroxide ions at high concentrations.We took the advantage of additive manufacturing by developing flow cells with integrated millifluidics channels for electrolyte circulation. These type of shapes cannot be made by classical manufacturing processes. Stereolithography has the advantage of freeing itself from several manufacturing constraints inherent in other manufacturing processes. The input of the flow system is divided into 3 channels. Each of these 3 channels has a cross-section 2 mm x 4 mm, and a length of 5 mm. At the output, 3 channels identical to those of the input are combined into one output channel. The output and input channels are connected to a tank of electrolyte by tubing (PharMed BPT, Saint-Gobain) with an internal radius of 3 mm and a length of 30 cm. The electrodes lies between the input zone and the output zone (see the orange zone in Fig. 10). Their composition is displayed below. Bubble-free electrolyte from the lower part of the tank is injected into the system inlet, whilst the electrolyte with oxygen bubbles from the cell is evacuated from the system outlet and injected into the upper part of the tank using a peristaltic pump (VWR PP3300). The flow rate was varied between 0 and 1.5 mL s^−1^. Under these conditions, the flow remains laminar and no turbulence occurs. To characterize this feature, we calculated the Reynolds number which is the ratio between the inertial forces and the viscous forces, $Re=\frac{\rho U h}{\eta }=\frac{\rho Q}{w \eta }$ where $\rho$ is the fluid density, U the mean velocity, *h* the distance between the two plates, w the width of the spacer and Q the flow rate. With $w=1.5$ cm, $\eta$ = 2.5 10$^{_3}$ Pa s, $\rho$ = 1000 kg m$^{-3}$, this leads to Reynolds number between 0 and 40 which corresponds to laminar flow.

#### Zinc Cell

The electrodes are fixed in the zone between the inlet an the outlet (see orange zone in Fig. 10). The negative zinc electrode is a plate which is 0.25 mm thick, 2.5 cm long and 1.5 cm wide. The positive OER nickel grid electrode has a mesh size of 550 microns and is 2.5 cm long and 1.5 cm wide.

Inside this Zinc flow cell, the zinc electrode is covered by a non-selective porous membrane (Celgard 4001) with 41% porosity and a thickness of 25 $\upmu$m. This type of membrane is classically used in Zinc batteries as a separator to prevent a short-circuit between the electrodes. An additional home made separator fixes the distance between the two electrodes at 2 mm. Figure 1 illustrates the Zinc-cell setup and its different components.

#### Platinum-Carbon set up

The electrodes are fixed in the zone between the inlet an the outlet (see orange zone in Fig. 10). The platinum and carbon electrodes are plates (GoodFellow). Electrodes are 0.25 mm thick, 2.5 cm long and 1.5 cm wide. A home made separator fixes the distance between the two electrodes at 2 mm. The configuration of the cell is the same as for the Zinc-cell setup shown in Fig. 1, replacing zinc anode and separator by the carbon electrode and the nickel OER electrode by the platinum electrode.

### Optical measurements

**Figure 11:**
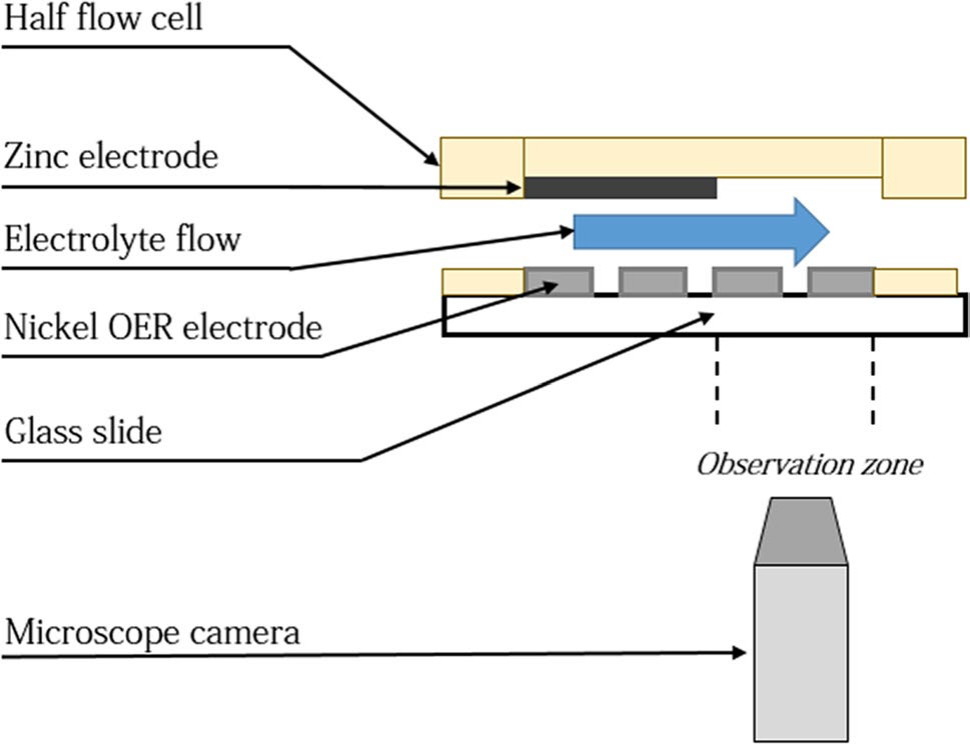
**S**chematic view of the microscope observations set-up. 1: Acquisition system, 2: Camera, 3: Mirror, 4: Microscope lens, 5: Electrochemical cell, 6: Scattered light.

Home-made cells designed for optical characterisation are held at the focal length of a microscope lens (magnification: ×1.25), the microscope itself is connected to a digital microscope camera (Magnification ×10, 10 frames per seconds) (Dinolite). The counter part of the cells presented in figure ?? is replaced by a glass slide to allow the optical measurements (see Fig. 11). The setup is connected to the peristaltic pump and the current generator. This device makes it possible to carry out experiments under the microscope for several flow rates and several current densities. The counter electrode (Zinc plate) is positioned so as not to obstruct bubble visualisation at the Nickel grid. The same observations were also performed for the Platinum/carbon system, Platinum is the positive electrode for OER in this situation. Given that it is a full, solid plate and not a grid, we are not able to monitor the formation of the oxygen bubbles on this electrode, we just characterize the size of the bubbles after their departure from the Platinum plate.

### Electrochemical characterization

The electrochemical behaviour of each electrode in our 2 systems was studied using a CH Instrument 600E potentiostat. The nickel electrode or the platinum electrode are setup as the working electrode whilst the zinc electrode or the carbon electrode are counter electrodes.

#### Cyclic Voltammetry

The systems were characterised by cyclic Voltammetry at a scan rate of 50 mV.s^−1^. In all experiments, a silver chloride Ag/AgCl (KCl sat.) reference electrode was used to measure the potential of the working electrode.

#### Chronopotentiometry

A constant current is caused to flow between the working electrode and the counter electrode. The potential changes of the working and counter electrodes are recorded with respect to the reference electrode.

The chronopotentiometry experiments are performed in absence and in presence of the flow. We used flow rates between 0 mL s^−1^ and 1.5 mL s^−1^, these corresponds to Reynolds numbers range between 0 and 24. Each experiment with electrolyte flow is made following a well defined protocole. First a current is applied in absence of flow. The potential of both electrodes is simultaneously recorded for 10 min, then the current is interrupted and the remaining bubbles are swept by using the electrolyte flow (Q = 1.5 mL s^−1^) for 10 s. This allows us to sweep the bubbles and start the measurement with a clean surface. We anticipate that a few bubbles may remain on the surface even with the use of flow. We will see in the following that this may cause a slight discrepancy between the modeling and the data. The interruption of the previous flow (ie Q=1.5 mL s^−1^) or a change of this flow used to remove bubbles combined with the start of the current marks the beginning of the next experiment. The following currents were studied: 3, 5, 7.5, 10, 20 mA cm^−2^.

### Measurements of the viscous losses

**Figure 12:**
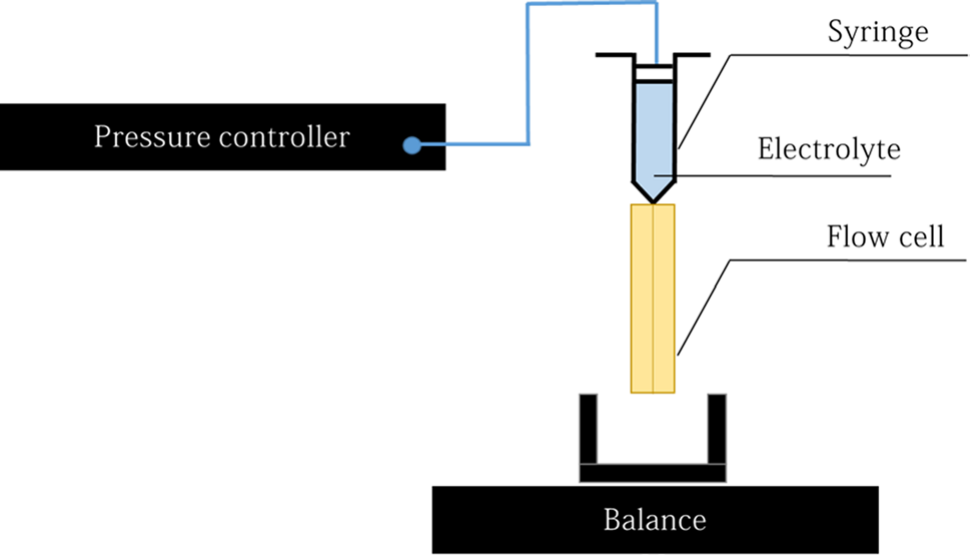
Scheme of the experimental set up used to measure the viscous losses.

In order to measure the viscous losses related to the flow in the cell, we carried out pressure drop measurements as a function of the flow rate (see Fig. 12). For this we connect the cell with a syringe and applied a pressure drop $\Delta P_{global}$ between the top of the syringe and the outlet of the cell thanks to a pressure controller (Fluigent). The maximal applied pressure by the controller is 700 mbar and the precision over the full range of measurement is equal to 10 Pa according to the manufacturer’s specifications. We set the pressure at the inlet of the cell and the outlet is at atmospheric pressure. To measure the flow rate, we weight the mass of electrolyte which flow from the cell with a precision scale. The measurements are averaged over 5 min. These experiments are performed in presence or absence of the electrical current in order to probe the influence of the bubbles over the hydrodynamic losses.

We get a relation between the global pressure drop $\Delta P_{global}$ and the flow rate. In order to separate the contribution of pressure drop upstream of the cell (i.e in the syringe and in the tubing) $\Delta P_{st}$ and of the one occurring in the cell itself $\Delta P$, the same experiments are carried out in the absence of the cell. We measure the relation between the pressure drop in the syringe and in the tubing $\Delta P_{st}$ and the flow rate. The pressure drop in the cell $\Delta P$ is deduced by making the difference between the two measurements ($\Delta P=\Delta P_{global}-\Delta P_{st}$). The flow rate *Q* is measured by weighing the quantity of fluid leaving the cell after 5 min of flow. These measurements are carried out in the presence and absence of electric current in order to probe the role of bubbles in viscous losses. The power of viscous losses in the cell $\mathcal {P}_{h}$ corresponds to the product $\mathcal {P}_{h}=\Delta P Q$.

## Supplementary Information

**Supplementary Information 1**.
