# Supplementary material for: Effect of electrolyte flow on a gas evolution electrode
Source: Sci Rep. 2021 Feb 25;11:4677. doi: 10.1038/s41598-021-84084-1 (PMC7907386; doi:10.1038/s41598-021-84084-1)
Supplement: Supplementary file 1 — Supplementary Information 1. [file 41598_2021_84084_MOESM1_ESM.pdf]

Supplementary materials:Effect of electrolyte flow on a gas evolution electrode

Soufiane Abdelghani Idrissi[1], Nicolas Dubouis[2], Alexis Grimaud[2], Philippe Stevens[3], Gwenaëlle Toussaint[3], and Annie Colin[1]

[1]ESPCI Paris, PSL Research University,MIE-CBI, CNRS UMR 8231, 10, Rue Vauquelin, F-75231 Paris Cedex 05, France.
[2]Chimie du Solide et de l'Energie, Collège de France, UMR 8260, 75231 Paris Cedex 05, France Sorbonne Université,Paris,France. Réseau sur le Stockage Electrochimique de l'Energie (RS2E), CNRS FR 3459,F-75005 80039 Cedex, Amiens, France,
[3]EDF R&D, EDF Lab Renardières, Département LME, 7 avenue des Renardières, 77818 Moret-sur-Loing cedex, France. Réseau sur le Stockage Electrochimique de l'Energie (RS2E), CNRS FR 3459,F-75005 80039 Cedex, Amiens, France,

December 22, 2020

1 Voltammetry experiments

1.1 Platinum electrode

The main reaction visible on the cyclic volatmograme of the platinum electrode (Fig.3), is the oxidation/reduction of the hydroxide. This suggests that the OER is the only oxidation reaction that needs to be taken into account. The oxidation reaction starts around 0.6 V vs (Ag/AgCl).

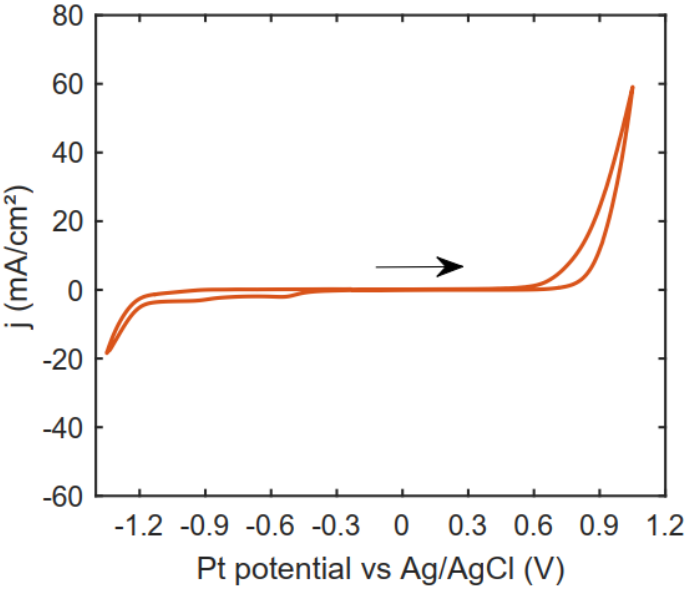

Figure S1: Voltammetry of the Platinum electrode versus Ag/AgCl electrode. The scan rate is equal to 50mV/s. The OER occurs at electrode potential around 0.7 V vs Ag/AgCl.

1.2 Nickel electrode

On the cyclic voltammetry of nickel electrode (see Fig.S2), we clearly see oxidation and reduction peaks compared to platinum. The first peak is observed around 0.5V vs Ag/AgCl. It is characteristic of the change in oxidation state from Ni(II) to Ni(III), where β-Ni$(OH)_2$ is oxidized to β-NiOOH. The electro-

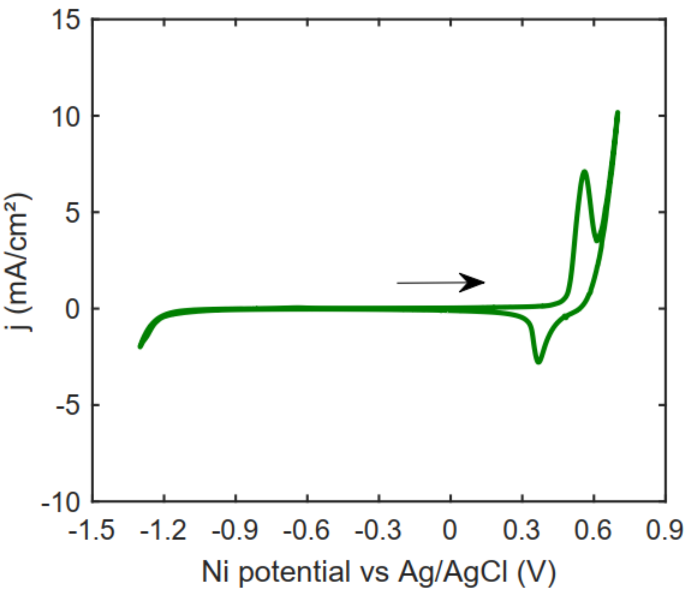

Figure S2: Voltammetry of the Nickel electrode versus Ag/AgCl electrode. The scan rate is equal to 50mV/s. The OER occurs at electrode potential close to 0.6 V vs Ag/AgCl.

chemical equations is:

$$\beta - Ni(OH)_2 + OH^- \rightarrow \beta - NiOOH + H_2O + e^- \quad (1)$$

Following potential increase, the next increase of the oxidation current occurs at approximately 0.6V vs Ag/AgCl and is attributed to the oxygen evolution reaction. In the cathodic region, β-NiOOH is reduced to $\beta-$Ni(OH)$_2$ at electrode potential close to 0.4V vs Ag/AgCl. This peak corresponds to a reversible reaction, the reversible potential is around 0.45V vs Ag/AgCl. At that potential, the OER may have already started and there is a mixture a both reactions. The reactions occurring at the electrode are the reversible reaction of Ni(II) to Ni(III) and OER as shown by the green line.

1.3 Carbon electrode

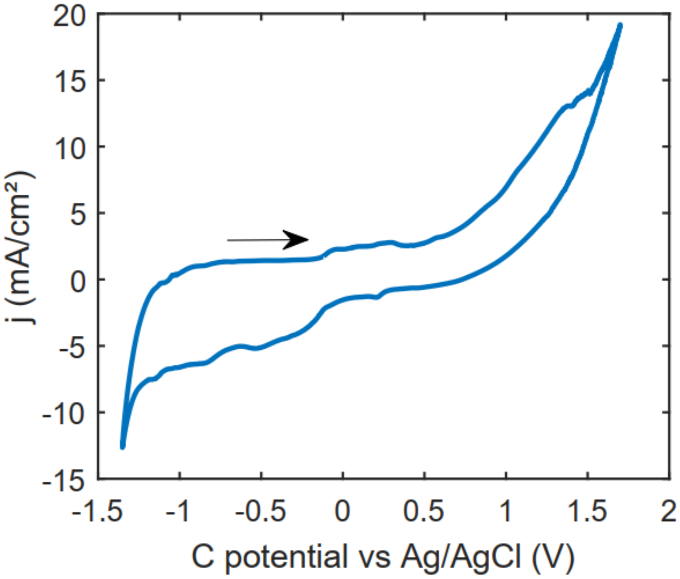

Figure S3: Voltammetry of the Carbon electrode versus Ag/AgCl electrode. The scan rate is equal to 50mV/s. The HER occurs around -1.15 V vs Ag/AgCl.

At the carbon electrode, the cathodic and anodic current are attributed to the oxidation and reduction of Carbon and degradation products at its surface. Carboxyl groupe are formed on the carbon surface for positive currents. This oxidation can lead to the degradation of the electrodes because of the loss of the graphitic surface layer when the layer becomes hydrophilic by the addition of carboxyl groups. At negative currents OH and OOH groups are reduced and there is indeed no hydroxide formation during HER.

1.4 Tafel plots

The Tafel plots in Fig S4 gives parameters needed in the modelling section.

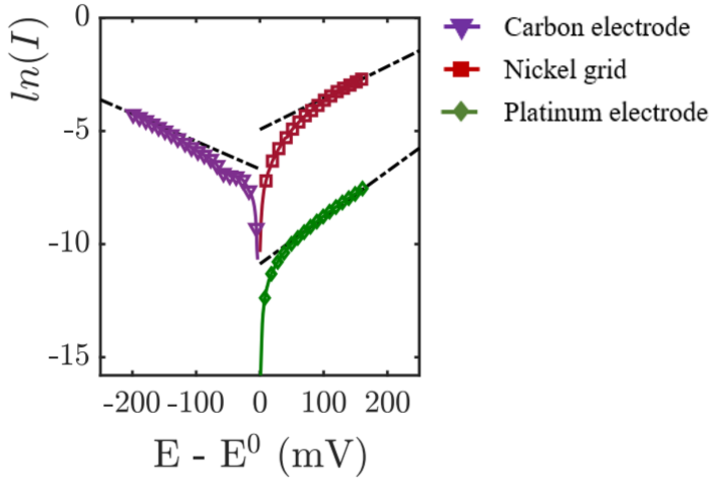

Figure S4: Tafel plot for the calculation of the electrochemical paramaters used in the model. Red squares : OER reaction at Nickel electrode ; Green diamonds : OER reaction at Pt electrode ; Purple triangles : HER reaction at carbon electrode.

2 Chronopotentiometry experiments

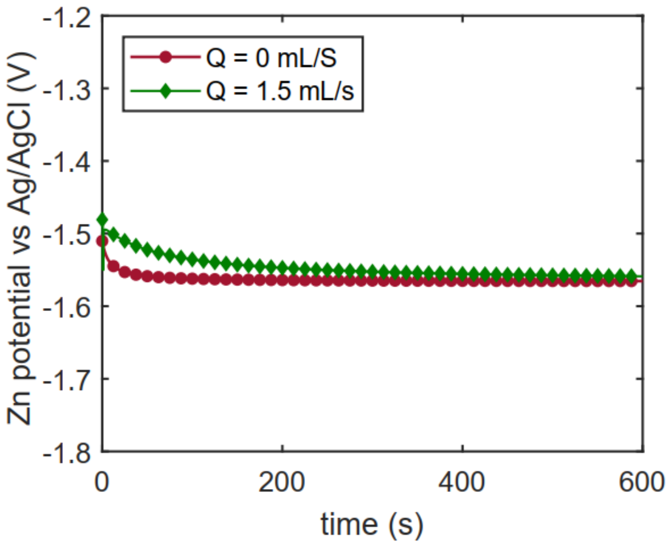

Figure S5: Potential evolution of the zinc electrode at j = 10 mA/cm^2. We observe a slight effect of the flow.
